# Anti-IgLON5 disease presenting with myokymia and favourable response to FcRN-antagonist combined with oral glucocorticoids: case report

**DOI:** 10.3389/fimmu.2026.1841316

**Published:** 2026-06-29

**Authors:** Tianling Shang, Bo Ma, Shumei Wen, Zuohong Tang, Xiaoliang Mao, Keping Jiao

**Affiliations:** 1 Department of Neurology, Gansu Provincial Hospital, Lanzhou, Gansu, China; 2 Department of Critical Care Medicine, Lanzhou University First Hospital, Lanzhou, Gansu, China

**Keywords:** anti-IgLON5 disease, FcRn antagonist, glucocorticoids, immunosuppression, myokymia

## Abstract

Anti-IgLON5 disease is a rare autoimmune neurological disorder characterized by anti- neuronal surface autoantibodies targeting the Immunoglobulin-Like Cell Adhesion Molecule 5 (IgLON5) protein. We report a 53-year-old male patient diagnosed with anti-IgLON5 disease who presented with the uncommon manifestation of generalized multi-site myokymia. Laboratory tests showed positive anti-IgLON5 IgG antibodies in both serum (titer 1:100) and cerebrospinal fluid (CSF) (titer 1:1), with no specific abnormalities detected on brain magnetic resonance imaging (MRI) and whole-exome sequencing. Considering patients’ refusal to use high-dose glucocorticoids due to concerns about drug side effects, as well as the uncertainty surrounding the efficacy of intravenous immunoglobulin in treating IgLON5 disease, we selected a combined immunosuppressive therapy regimen consisting of intravenous neonatal fragment crystallizable receptor (FcRn) antagonist (efgartigimod) and sequential oral prednisone. The patient’s myokymia were rapidly alleviated after combined treatment. During an 18-month follow-up, symptoms completely abated within the first 6 months, and antibody titers declined gradually to negative. Mild symptom recurred at 7 months post-initial treatment. Serum anti-IgLON5 IgG antibodies became positive (titer 1:10) at 14 months, however, these antibodies had been negative at 3 months after the start of treatment. Symptomatology was well-controlled after adjusting the oral prednisone dose. This case expands the clinical spectrum of anti-IgLON5 disease, highlights the necessity of screening for neuronal autoantibodies in patients with myokymia, and suggests that the combination of FcRn antagonists and glucocorticoids may emerge as a new immunotherapeutic option for this condition.

## Introduction

Anti-IgLON5 disease is a recently defined autoimmune neurological disorder with diverse clinical manifestations, complex pathogenic mechanisms, and notable diagnostic and therapeutic challenges (1). Its primary clinical phenotypes include bulbar, movement disorder, sleep, cognitive, and neuromuscular subtypes (2).

Neuromuscular symptoms encompass muscle weakness, muscle atrophy, myoclonus, fasciculation, myorhythmia, fibrillation, and myokymia, among others (2). Myokymia, presented as persistent, slow, wave-like superficial muscle contractions, is relatively infrequent and prone to being misdiagnosed. In contrast to other autoimmune encephalitides (AEs) associated with neuronal surface antigen antibodies, anti-IgLON5 disease typically exhibits a suboptimal response to immunotherapy, and standardized treatment protocols remain lacking (3). Herein, we report the rare case of anti-IgLON5 disease manifesting predominantly as myokymia that achieved a favorable therapeutic response to an FcRn antagonist combined with sequential oral glucocorticoids.

## Case presentation

A 53-year-old male was admitted to our hospital in July 2024 with an 8-month history of intermittent generalized myokymia, which had exacerbated over the preceding month. The symptom initially exhibited localized myokymia in the right upper limb, occurring at rest, alleviating with activity, and worsening with emotional arousal or stress; each episode lasted several seconds and resolved spontaneously. Subsequently, the clinical presentation progressed, with the affected area extending from the right upper limb to both upper limbs, back, lumbosacral region, anterior chest, and both lower limbs, and the frequency increasing to more than ten episodes daily. (**Video 1**)

One month prior to admission, the patient developed anxiety related to his son’s college entrance examination, which was accompanied by a significant exacerbation of myokymia. During the clinical course, the patient denied insomnia, and no muscle atrophy, pain, limb numbness, or weakness was noted. Prior treatment with oral baclofen and acupuncture yielded poor efficacy. The patient had a history of hypertension, no smoking or alcohol consumption, no drug or food allergies, and no family history of relevant neurological disorders.

Vital signs were normal on admission. Neurological examination revealed fasciculations of the tongue muscles, and persistent, wave-like myokymia was observed in the limbs and thoracic back region. The myokymia was migratory in nature, predominantly affecting the limb and trunk skeletal muscles in a random shifting pattern. It was more prominent at rest and unrelieved by voluntary activity. Neurological assessment presented normal cognitive function (Montreal Cognitive Assessment (MoCA) score 26/30, with a college education or higher). No other neurological deficits were identified. At this time, the IgLON5 disease clinical score (ICS) was 5 points, which is a validated indicator of disease severity (4).

Limb electromyography (EMG) demonstrated resting fasciculation potentials in several examined muscles (vastus lateralis, vastus medialis, gastrocnemius, dorsal interossei, and deltoid), with no neurogenic or myogenic damage observed during mild or maximal voluntary contraction. Brain MRI indicated scattered punctate T1-hypointense, T2-hyperintense, and FLAIR-hyperintense lesions in the bilateral frontal and parietal subcortical and periventricular regions, without abnormal enhancement or diffusion restriction, suggestive of white matter demyelination. Whole-spine MRI revealed reduced T2WI signal of cervical and lumbar intervertebral discs, C5/6 disc bulging, C6/7 disc herniation, and L3/4, L4/5, and L5/S1 disc herniation. (**Figure 1**) Polysomnography and ambulatory electroencephalography were unremarkable.

**Figure 1:**
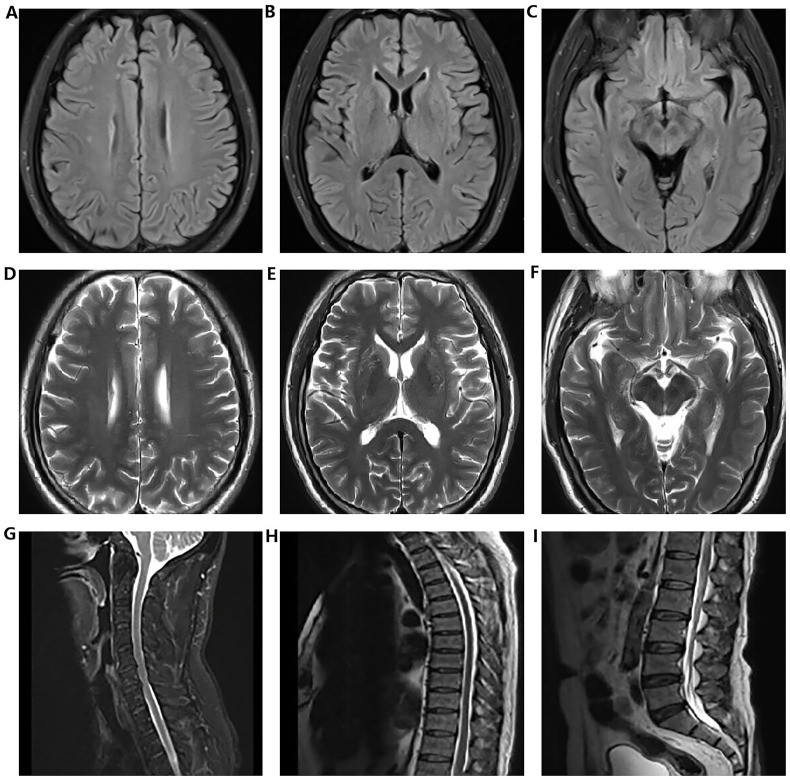
**(A–C)** Brain MRI images show scattered punctate FLAIR hyperintensities in the bilateral periventricular regions, centrum semiovale, and frontal lobes; **(D–F)** Scattered punctate T2 hyperintensities in the bilateral periventricular regions, centrum semiovale, and frontal lobes; **(G–I)** Cervical, Thoracic, and Lumbar Spine FLAIR Sequence.

Laboratory investigations reflected elevated serum creatine kinase (CK: 666.71 U/L; reference range: 50–310 U/L), positive antinuclear antibodies, and slight elevation of cytokeratin 19 fragment (a tumor marker); follow-up testing confirmed normalization of serum CK. CSF analysis manifested increased intracranial pressure (240 mmH2O), mild hyperproteinemia (0.53 g/L), and normal white blood cell count, IgG, glucose, and chloride levels. Anti-IgLON5 IgG antibodies were positive in both serum (titer 1:100) and CSF (titer 1:1). (**Figure 2**) Serum paraneoplastic autoantibodies and peripheral neuropathy-related antibodies were all negative. Whole-exome sequencing failed to detect the HLA-DRB110:01-DQB105:01 haplotype. Of note, formal HLA typing was not performed in this patient.

**Figure 2:**
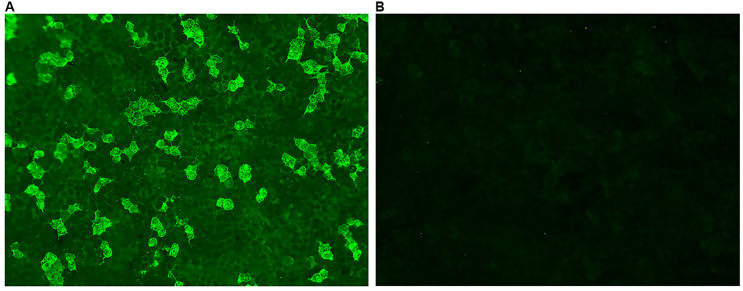
IgLON5-expressing HEK293T cells were immunofluorescence-stained with serum and CSF. **(A)** Anti-IgLON5 IgG titer in the serum was 1:100 (×40). **(B)** Anti-IgLON5 IgG titer in the CSF was 1:1 (×40). CSF, cerebrospinal fluid.

Based on the subacute onset, major neuromuscular symptoms, and positive anti-IgLON5 antibodies in both serum and CSF, the patient met the diagnostic criteria for anti-IgLON5 disease (5). The patient lacks typical clinical phenotypes such as myasthenia, myotonia, and painful spasms. Electromyography did not reveal persistent rhythmic discharges, and peripheral neuropathy antibody tests were negative, allowing the exclusion of peripheral nerve hyperexcitability disorders (e.g., Isaacs syndrome, neuromyotonia) (6). The patient has no symptoms of myasthenia, muscle atrophy, or bulbar palsy. Electromyography did not show neurogenic damage, and there was no progressive neurological deficit during the 8-month course, thus excluding motor neuron disease (7). The patient’s paraneoplastic antibody tests were normal, and there were no seizures, cognitive decline, or psychiatric symptoms, allowing the exclusion of other AEs (8). The patient declined high-dose glucocorticoid pulse therapy due to concerns regarding potential adverse effects. Thus, a comprehensive immunosuppressive regimen was formulated with intravenous efgartigimod alfa (10 mg/kg weekly for 4 consecutive weeks) followed by sequential oral prednisone (initial dose: 60 mg daily, tapering by 5 mg weekly).

The follow-up lasted 18 months, during which the disease course could be divided into three phases: acute treatment, maintenance, and relapse with retreatment. One month after the initial administration of efgartigimod, the patient’s myokymia improved significantly, the ICS score was reduced from 5 to 3, the serum anti-IgLON5 IgG titer decreased to 1:32, and CSF antibodies turned negative. Given the persistent positive serum antibodies, oral prednisone was initiated as planned (60 mg daily, tapering by 5 mg weekly). At the 3-month follow-up, clinical symptoms had completely resolved, the corresponding ICS score has been declined to 2 points, and both serum and CSF anti-IgLON5 antibodies were negative. Subsequent maintenance therapy continued with oral prednisone administration. When the prednisone dose was tapered to 10 mg daily (7 months post-initial immunotherapy), mild symptoms reappeared, the ICS score subsequently increased to 4 points, and this was controlled by adjusting the prednisone dose to 20 mg daily. The patient consequently discontinued the medication without medical advice. At 14 months post-diagnosis, myokymia exacerbated considerably accompanied by moderate anxiety. The ICS score has risen again to 5 points, the pre-treatment level, and serum anti-IgLON5 IgG antibodies were positive (titer 1:10). Prednisone was reinitiated at 60 mg daily and tapered by 5 mg weekly to a maintenance dose of 20 mg daily. A telephone follow-up in February 2026 displayed a marked reduction in the range and frequency of myokymia compared with the relapse phase (**Video 2**), the ICS score decreased to 3 points (**Table 1**). No infections or other drug-related adverse events were observed throughout the entire treatment and follow-up period.

**Table 1:** The timeline of the entire treatment process, including medication administration, changes in clinical symptoms, laboratory data, and ICS scores.

Category		Timeline						Units	Normal range
		2024.07	2024.08	2024.10	2025.02	2025.09	2026.02		
Clinical symptom	Myokymia	+++	+	–	++	+++	+	N/A	N/A
	Cognitive impairment (MOCA)	26	26	26	/	26	/	score	26-30
	Other symptoms	–	–	–	–	–	–	N/A	N/A
CSF findings	Intracranial pressure	240	150	140	/	200	/	mmH2O	80-180
	WBCs	2	2	2	/	0	/	10^6^/L	0-8
	Protein	0.53	0.74	0.48	/	0.47	/	g/L	0.15-0.45
	IgG	54.3	27.5	21.2	/	45.9	/	mg/L	4.8-58.6
	Anti-IgLON5 IgG	1:1	–	–	/	–	/	N/A	–
Anti-IgLON5 IgG in serum		1:100	1:32	–	/	1:10	/	N/A	–
ICS		5	3	2	4	5	3	score	0

ICS, anti-IgLON5 disease composite score; WBCs, white blood cells; CSF, cerebrospinalfluid; MRI, magnetic resonance imaging; -, +, ++, +++ represent negative, mild, moderate, and severe respectively; /, no record available. N/A, not applicable.

## Discussion

First described in 2014 as a unique sleep disorder (9), anti-IgLON5 disease has since been recognized to have a broad clinical spectrum, most commonly including brainstem dysfunction, gait abnormalities, movement disorders, autonomic nervous system dysfunction, oculomotor abnormalities, neuromuscular symptoms, and neurocognitive or psychiatric changes. These manifestations may occur in isolation or in various together with sleep disturbances (2, 5). We report an exceptional case presenting with isolated myokymia, an atypical movement disorder caused by peripheral nerve hyperexcitability or abnormal motor unit discharge, which is also observed in other neurological disorders including AEs, peripheral neuropathies, motor neuron disease, metabolic neuropathies, and drug-related adverse reactions (10). In anti-IgLON5 disease, cases characterized by myokymia as the sole primary symptom are extremely rare. The patient’s clinical feature of rippling-like muscle movements in the limbs was consistent with the classic features of myokymia (11). Spontaneous rhythmic burst discharges are the typical electrophysiological hallmark of myokymia, however, only resting fasciculation potentials were detected on the patient’s EMG, with no continuous rhythmic discharges. This finding may be connected with the paroxysmal nature of the patient’s myokymia and the interictal timing of the EMG examination.

Brain MRI FLAIR hyperintensities were noted in the patient, with no obvious changes before and after treatment. Therefore, these punctate lesions were considered nonspecific white matter demyelination and not clearly associated with anti-IgLON5 disease. Minor CSF hyperproteinemia was observed on routine analysis, in accordance with previous studies (12) but lacking diagnostic specificity. Studies have confirmed a strong correlation between the human leukocyte antigen haplotype HLA- DRB1*10:01-DQB1*05:01 and anti-IgLON5 disease (13). Nevertheless, this genetic marker was not detected in our patient, which seems to align with Graus et al.’s research (12) showing that only 59% of anti-IgLON5 disease patients carry the haplotype, with a higher prevalence in those exhibit sleep disturbances and bulbar symptoms. Our patient had no sleep disorder, and polysomnography was unexceptional. A definitive diagnosis of anti-IgLON5 disease was made rely on the primary presentation of myokymia and positive anti-IgLON5 antibodies in both serum and CSF (1). Collectively, the patient’s clinical phenotype, sleep monitoring, and cranial imaging findings were inconsistent with the typical features of anti-IgLON5 disease, which may be related to the early stage of the disease course. Typical clinical features such as sleep disturbances and brainstem dysfunction may emerge as the disease progresses, highlighting the importance of neuronal autoantibody screening in patients manifested as isolated myokymia.

Unlike other AEs linked to neuronal surface antigen antibodies, anti-IgLON5 disease typically follows a chronic progressive course, with fewer than 40% of patients achieving a prominent response to immunotherapy (3). This may be attributed to unclear underlying pathophysiological mechanisms, including whether the disease is primarily mediated by autoimmunity or neurodegeneration (14, 15). Currently, there is no standardized treatment for anti-IgLON5 disease. A systematic review (16) of immunotherapy responses in anti-IgLON5 disease illustrated that combination therapy was more likely to yield a positive response than monotherapy, and second-line therapy had a higher response rate than first-line therapy. Nonetheless, the most effective immunotherapeutic agent or combination regimen for inducing remission or modifying the progressive course remains unclear, as does whether all clinical symptoms respond equally to immunotherapy. In spite of this, initiation of long-term immunotherapy within the first year of onset is a well-established predictor of a favorable prognosis (3).

Anti-IgLON5 disease is predominantly implicated in autoantibodies targeting the IgLON5 protein, which are of the IgG type, including IgG1, IgG2, and IgG4. Most patients harbor IgG1 and IgG4 antibodies (5). IgG1 can induce irreversible internalization of IgLON5, leading to reduced cell surface expression (17), while IgG4 mainly interferes with the interactions of the IgLON5 protein (18). Studies (5) have also revealed that IgLON5 undergoes ectodomain shedding and interacts with other members of the IgLON family, participating in biological processes such as neural plasticity, axon guidance, or cell migration. Both IgG1 and IgG4 antibodies can disrupt these interactions. Additionally, research (19) using human induced pluripotent stem cell (iPSC)-derived neuron-antibody models has demonstrated that treating neurons with patient-derived IgG results in downstream signaling events from antibody-neuron interactions, consisting of cytoskeleton disruption and calcium homeostasis disturbance. Another neuronal cell study (20) also indicated that regardless of the ratio of IgG1 to IgG4, it leads to changes in the cytoskeleton. In summary, anti-IgLON5 antibodies exert pathogenicity through direct or indirect mechanisms.

The patient refused high-dose intravenous methylprednisolone pulse therapy (the classic first-line treatment for AEs), and existing studies have reported conflicting results regarding the efficacy of intravenous immunoglobulin (IVIg) in anti-IgLON5 disease (21, 22). In light of the current treatment landscape and the aforementioned antibody pathogenicity, we selected a comprehensive immunosuppressive regimen coupling an FcRn antagonist with sequential oral prednisone tablets. This combined therapy was implemented in three phases: acute symptom control, remission maintenance, and retreatment for relapse. During the acute phase, a single cycle of monotherapy with efgartigimod resulted in considerable improvement in myokymia, a reduction in the ICS, a decrease in serum anti-IgLON5 IgG titer from 1:100 to 1:32, and negative CSF antibodies. As an FcRn antagonist, efgartigimod exerts its core therapeutic effect by competitively blocking the FcRn-IgG interaction, inhibiting IgG recycling, and accelerating lysosomal degradation of IgG, thereby rapidly reducing peripheral circulating IgG and pathogenic antibody titers (23). Due to its large molecular weight, efgartigimod cannot actively cross the intact blood-brain barrier (BBB) to directly clear CSF antibodies (24). Consequently, we hypothesized that the CSF antibody negativity may not result from direct drug entry into the brain for clearance but was implicated in two factors: First, reversal of the systemic immune microenvironment. Decreased serum antibody levels may alleviate peripheral immune pressure, which could inversely inhibit plasma cell activity within the central nervous system (CNS), accordingly interrupting the sustained source of CSF antibodies (25). Second, repair of the BBB and antibody reflux. This case suggests that after immune inflammation is controlled, the damaged BBB may be repaired. Restoration of BBB tight junctions would, on one hand, block re-entry of peripheral antibodies into the CNS and, on the other hand, promote unidirectional reflux of remaining antibodies in the CSF back into the circulation, ultimately leading to a rapid decline in CSF antibody titers to negative (24). Furthermore, the initial CSF anti-IgLON5 IgG titer in our patient was low (1:1), which likely stem from the rapid achievement of negative CSF antibodies after one month of treatment via the aforementioned dual mechanisms; patients with higher CSF antibody titers may require a longer treatment course. Efgartigimod served as the crucial agent in the acute phase, initiating the reduction of serum antibodies and the negativity of CSF antibodies. Succeeding sequential oral prednisone treatment resulted in complete resolution of myokymia and negative serum antibodies at 3 months. This therapeutic response further displays the central role of prednisone in controlling CNS immune inflammation and consolidating treatment efficacy: prednisone inhibits the NF-κB pathway and T/B cell activation to block pathogenic antibody synthesis at the source, and exerts potent anti-inflammatory effects to repair the damaged BBB and prevent peripheral antibody entry into the CNS (26–28). A study of efgartigimod in myasthenia gravis revealed that the maximal therapeutic effect occurs 3–5 weeks after initiation and persists for up to 8 weeks (29). In consequence, the distinct improvement in acute phase symptoms and the negativity of serum antibodies may depend both on the anti-inflammatory and barrier-repairing effects of glucocorticoids and the duration of efgartigimod’s efficacy. During the maintenance and retreatment phases, oral prednisone was the vital agent for sustained immune control: mild relapse occurred when the dose was tapered to 10 mg daily, indicating an insufficient maintenance dose to suppress immune activity, and symptom control was re-established after dose adjustment. Following self-discontinuation of prednisone and subsequent relapse at 14 months further reflected the irreplaceable role of prednisone in maintaining long-term remission. Reinitiation of an adequate prednisone dose and gradual tapering to a maintenance dose of 20 mg daily resulted in rapid symptom relief, demonstrating that prednisone is the major agent for clinical control in both the acute phase and relapse retreatment. Concurrently, we observed that the ICS score exhibited fluctuating changes as the treatment progressed. The score decreased when symptoms were alleviated and increased when symptoms recurred. To summarize, the marked clinical outcome in this case cannot be ascribed to a single agent but reflects a sequential synergistic, phase-dominant therapeutic effect: efgartigimod primarily reduces the peripheral antibody burden to exert an acute-phase “debulking” effect, while prednisone is the primay agent for controlling CNS inflammation, blocking antibody synthesis, and maintaining long-term remission. Considering the generally suboptimal immunotherapy response in anti-IgLON5 disease, early administration of an FcRn antagonist to rapidly lower antibody levels followed by sequential glucocorticoids for long-term immune modulation may represent a feasible strategy to optimize clinical outcomes.

As a novel immunosuppressant, the safety profile of efgartigimod is a critical consideration for clinical application. Efgartigimod is the first globally approved FcRn antagonist for the treatment of anti-acetylcholine receptor (AChR) antibody-positive generalized myasthenia gravis (gMG) in adults (30), with well-documented safety, efficacy, and tolerability (31). Efgartigimod can reduce total peripheral circulating IgG levels by approximately 60–70% (32), and its high selectivity for IgG preserves other immunoglobulin subtypes (IgA, IgM, IgE, and IgD) (33). Although this IgG reduction theoretically increases the risk of infection, clinical studies have not observed an elevated incidence of severe infections (33). This beneficial safety profile is ascribed to two key features: First, the reduction in IgG levels is totally reversible after treatment discontinuation, with IgG levels returning to baseline approximately 8 weeks after the last dose and no permanent damage to the FcRn system (34). Second, specific humoral immune responses to vaccines are largely preserved, as efgartigimod only clears circulating IgG without depleting B cells or plasma blasts or inhibiting new antibody production (33). Throughout the 18-month follow-up, our patient developed no infections or other immunodeficiency-related disorders, further confirming the promising safety profile of efgartigimod compared with other immunosuppressants. Moreover, efgartigimod has been extensively investigated in IgG-mediated autoimmune diseases (35), including AEs such as anti-GABABR encephalitis, anti-LGI1 encephalitis, and anti-NMDAR encephalitis (36).This case further widens the clinical applicability of efgartigimod in autoimmune encephalitis, including anti-IgLON5 disease.

Current first-line and second-line treatments for AEs have evident limitations, including incomplete efficacy and concerning toxic burden (36). In this case, the novel immunosuppressant efgartigimod in combination with the traditional immunosuppressant glucocorticoids yielded an advantageous clinical outcome, providing new insights into the treatment of anti-IgLON5 disease. At the last telephone follow-up in February 2026, the patient’s myokymia was well-controlled, with no emergence of brainstem dysfunction (e.g., sleep disturbances, dysphagia, dysarthria, vocal cord paralysis) or other common manifestations (e.g., gait instability). Long-term follow-up of this patient will be continued to monitor for disease progression and long- term treatment efficacy.

Unexpectedly, we observed an association between anxiety and disease exacerbation. It remains unclear whether severe anxiety is a trigger for myokymia or one of the neuropsychiatric symptoms of anti-IgON5 disease itself. An experimental study using IgLON5 knockout mice found that IgLON5 is involved in the regulation of activity, anxiety, motor function, and contextual fear memory (37), suggesting that anxiety may result from loss of IgLON5 function. Another study (38) also demonstrated that IgLON5 deficiency is linked to psychiatric symptoms in animal models, supporting a potential direct relation between IgLON5 dysfunction and anxiety in anti-IgLON5 disease.

This study has several limitations: First, it is a single-center case report, lacking comparative analysis and external validation. Second, tau PET imaging was not optimized to definitively confirm the presence of pathological tau protein deposition in the brain, a known feature of advanced anti-IgLON5 disease. According to current literature, this is the first report of efgartigimod for the treatment of anti-IgLON5 disease with a critical therapeutic outcome. Notwithstanding, owing to the single-case design and concurrent use of glucocorticoids, the conclusions of this study are preliminary and require validation in large-scale, multi-center clinical studies. Future research should include multi-center randomized controlled trials to further explore the efficacy and underlying mechanisms of efgartigimod in the treatment of anti- IgLON5 disease.

## Conclusion

Myokymia, as an isolated neuromuscular symptom, significantly expands the clinical spectrum of anti-IgLON5 disease. Concurrently, this study identified that the novel FcRn antagonist efgartigimod integrated with conventional glucocorticoids represents a promising targeted therapeutic strategy for anti-IgLON5 disease, providing new clinical evidence for the management of this rare and challenging autoimmune neurological disorder.

## Data Availability

The datasets presented in this study can be found in online repositories. The names of the repository/repositories and accession number(s) can be found in the article/**Supplementary Material**.

## Supplementary material

Supplementary Video 1 Taking the local myokymia of the right upper limb as an example, the most severe sign was not captured. The capture time was July 2024.

Supplementary Video 2 Myokymia of the patient’s right upper limb was captured in February 2026, while Myokymia in other regions had disappeared.
